# An impressive case of Rowell syndrome with extensive mucosal involvement successfully treated with anifrolumab

**DOI:** 10.1016/j.jdcr.2023.12.020

**Published:** 2024-01-19

**Authors:** Daniel R. Antohi, Anitha Ramu, Tian Zhu, Shudan Wang, Michael Occidental, Bijal Amin, Benedict Wu, Jeanie Lee

**Affiliations:** a Division of Dermatology, Montefiore Medical Center, Albert Einstein College of Medicine, Bronx, New York; b Department of Rheumatology, Montefiore Medical Center, Albert Einstein College of Medicine, Bronx, New York; c Department of Pathology, Montefiore Medical Center, Albert Einstein College of Medicine, Bronx, New York

**Keywords:** anifrolumab, discoid lupus erythematosus, malar rash, Rowell syndrome, systemic lupus erythematosus, type 1 interferon

*To the Editor:* Rowell syndrome (RS) is a rare disease characterized by erythema multiforme-like lesions in patients with systemic lupus erythematosus (SLE) or cutaneous lupus.^1^ Therapeutic options for managing RS include hydroxychloroquine, chloroquine, azathioprine, dapsone, thalidomide, and corticosteroid therapy.^2^ While most patients with RS respond well to the aforementioned first-line therapies, reports suggest an urgent need for more effective treatment options for recalcitrant cases. For example, Sigh et al^3^ introduced rituximab as an effective treatment for refractory RS. We also greatly appreciate Shope et al^4^ reporting a uncommon patient with RS, without mucosal lesions, showing excellent response to anifrolumab, a human monoclonal antibody to the type 1 interferon receptor, after failing first-line treatments and rituximab.^4^^,^^5^ To our knowledge, this is the only case reported in the literature of successfully using anifrolumab for RS. Herein, we highlight another patient with RS with severe mucocutaneous lesions that resolved with anifrolumab after failing standard first-line treatments and belimumab.

A 41-year-old woman with SLE presented to the hospital with a 1-month history of gradually worsening painful eruptions affecting the lips, oral mucosa, palms, upper arms, and groin. She was diagnosed with SLE in 2012, manifested by malar rash, pericarditis, arthritis with serologies positive for antinuclear antibody 1:320 in a speckled pattern, double-stranded DNA, anti-Ro/SSA antibody, and low C3 and C4. Her clinical presentation worsened despite therapy escalation by her outpatient rheumatologist, which included prednisone, mycophenolate mofetil, hydroxychloroquine, and belimumab.

On examination, the patient reported a severe malar eruption with glabella involvement and erythematous scaly plaques involving the frontal and occipital scalp (Fig 1, *A*). The erythematous papules affected the columella with extension to the vermilion border (Fig 1, *B*). Red to violaceous targetoid macules, papules, and plaques affected the trunk, arms, and palms (Fig 1, *C*, *D*). The oral mucosa and labia majora had severe erosions and ulcerations.

**Fig 1:**
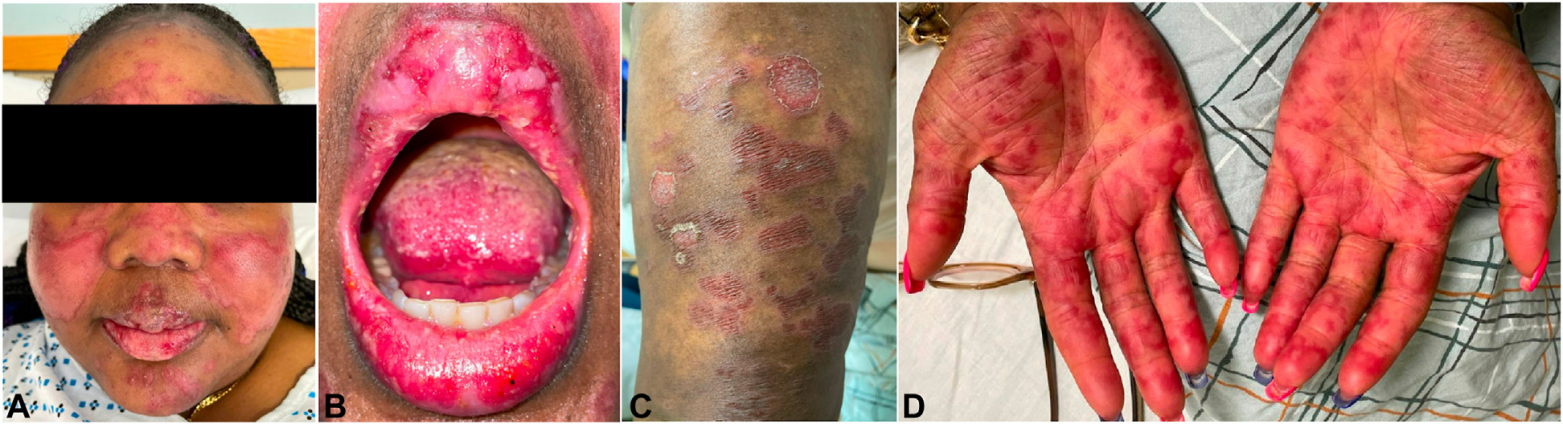
Initial presentation of Rowell syndrome (RS) lesions on the face **(A)**, the lips **(B)**, the left posterior arm **(C)**, and the palmar surfaces of the hands **(D)**. Note the *bright red* to violaceous papules, plaques, and erosions.

Laboratory testing showed positive anti-double-stranded DNA and anti-Ro antibodies, with low C3 and C4. Lesional skin biopsy revealed acute discoid lupus with extensive necrotic keratinocytes (Fig 2, *A*, *B*). The peri-lesional direct immunofluorescence study showed granular IgG, IgM, IgA, and C3 deposition in the basement membrane zone, consistent with the lupus band test. Rowell syndrome was diagnosed based on clinico-pathologic-serologic correlation.

**Fig 2:**
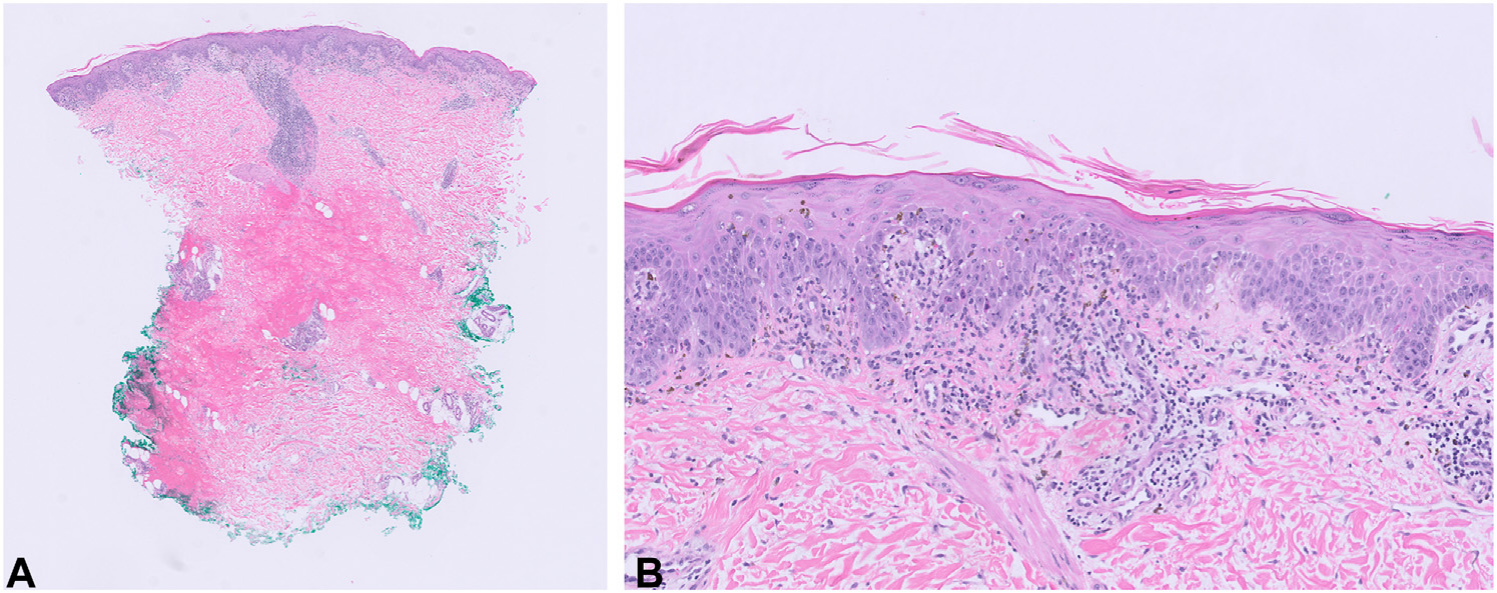
Histopathology of Rowell syndrome of the left posterior arm. **(A)** H&E, ×1. Lesional punch biopsy shows an inflammatory cell infiltrate within the papillary dermis and around adnexal structures **(B)** H&E, ×20. There is vacuolar interface dermatitis and numerous necrotic keratinocytes within the epidermis.

The patient was treated with intravenous methylprednisolone 40 mg twice daily, triamcinolone 0.1% ointment to the face and body, mupirocin 2%, and gentamicin 0.1% ointments to the erosions. Two weeks after discharge, the patient was started on anifrolumab, and her lesions continued to remit and remained inactive at the 3-month follow-up visit (Fig 3).

**Fig 3:**
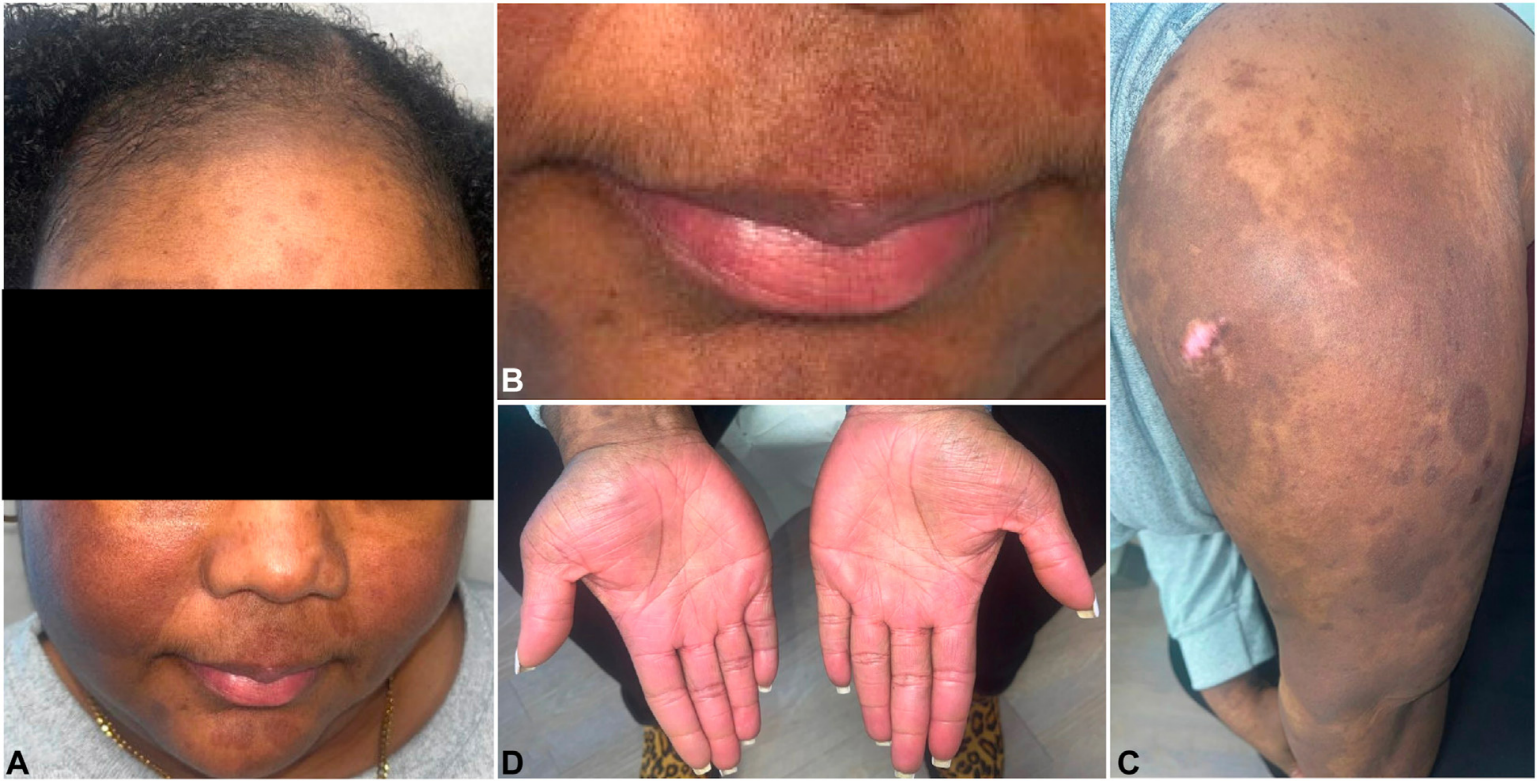
Complete clinical response to anifrolumab and hydroxychloroquine. Three-month post-treatment images of the face **(A)**, the lips **(B)**, the left posterior arm **(C)**, and the palmar surfaces of the hands **(D)**. For all the affected sites, the initial intensely *bright red* erythema and erosions healed with residual dyspigmentation and the absence of scarring.

We present this case intending to enrich the limited reports of RS and add to the previous report of anifrolumab for RS. Our case is unique because of the severe mucosal involvement at diagnosis. Given the patient’s excellent response to anifrolumab, we echo Shope et al^4^ recommendation of anifrolumab in refractory cutaneous lupus, especially when significant mucosal lesions are present. Further studies are warranted to examine the efficacy of anifrolumab for other cutaneous lupus variants.

## Conflicts of interest

None disclosed.
